# Transcriptomic Changes Underlying the Anti-Steatotic Effects of DHA Supplementation in Aged Obese Female Mice

**DOI:** 10.3390/ijms262311689

**Published:** 2025-12-02

**Authors:** Álvaro Pejenaute Martínez de Lizarrondo, Paula Martín-Climent, María Martínez-Rubio, Jesús Saborido-Gavilán, Neira Sáinz, Elisa Félix-Soriano, Miriam Samblas, Mónica Alfonso-Núñez, Elizabeth Guruceaga, M. Pilar Lostao, Pedro González-Muniesa, María J. Moreno-Aliaga

**Affiliations:** 1 Department of Nutrition, Food Science and Physiology and Center for Nutrition Research, School of Pharmacy and Nutrition, University of Navarra, 31008 Pamplona, Spain; apejenaute@alumni.unav.es (Á.P.M.d.L.); pmartinclim@external.unav.es (P.M.-C.); mmartinezru@alumni.unav.es (M.M.-R.); jsaboridoga@unav.es (J.S.-G.); nsainz@unav.es (N.S.); elisafelix93@gmail.com (E.F.-S.); msamblas@unav.es (M.S.); monalnu@hotmail.com (M.A.-N.); plostao@unav.es (M.P.L.); pgonmun@unav.es (P.G.-M.); 2 CIBER de Fisiopatología de la Obesidad y Nutrición (CIBEROBN), Instituto de Salud Carlos III, 28029 Madrid, Spain; 3 Instituto de Nutrición y Salud (INS), University of Navarra, 31008 Pamplona, Spain; 4 IdISNA—Navarra Institute for Health Research, 31008 Pamplona, Spain; eguruce@unav.es; 5 Bioinformatics Platform, CIMA, University of Navarra, 31008 Pamplona, Spain

**Keywords:** obesity, docosahexaenoic acid, metabolic dysfunction-associated steatotic liver disease, lipid metabolism

## Abstract

The prevalence of metabolic dysfunction-associated steatotic liver disease (MASLD) increases with age and obesity. The aim of this study was to characterize the transcriptomic changes in liver underlying the beneficial effects of docosahexaenoic acid (DHA) supplementation on MASLD progression in aged obese female mice. Two-month-old C57BL/6J female mice were fed ad libitum with high fat saturated diet (HFD, 45%) for 4 months. These diet-induced obese (DIO) mice were divided into two groups: the DIO group that continued with HFD and the DIO + DHA mice fed up to 18 months with the HFD containing a DHA-rich concentrate. High quality RNA from liver was used to perform RNA sequencing analysis. Further functional and clustering analyses of differentially expressed genes (DEGs) in liver between treatments revealed that DHA regulated processes related to lipid metabolic processes. DHA treatment also affects the PPAR signaling pathway and the regulation of inflammatory response. These data suggest that long-term DHA supplementation ameliorates MASLD progression in aged obese female mice by promoting transcriptomic changes in genes related to lipid metabolism and inflammatory response.

## 1. Introduction

Metabolic dysfunction-associated steatotic liver disease (MASLD) is the new name for non-alcoholic fatty liver disease (NAFLD), established by a multi-society Delphi consensus statement as a more appropriate term to correctly define the disease [1]. MASLD pathology is characterized by a broad spectrum of stages that ranges from simple steatosis, metabolic dysfunction-associated steatohepatitis (MASH), liver fibrosis, and cirrhosis to eventually hepatocellular carcinoma. Generally, most patients only suffer simple hepatic steatosis, which is characterized by fat accumulation in the liver without significant inflammation or fibrosis, and only a small fraction (5 to 10%) develop MASH [2], which is defined by liver lobular inflammation, severe steatosis, and hepatocyte ballooning [3]. The situation of chronic inflammation of MASH could result in the development of liver fibrosis, which is one of the main causes of death in MASH [4].

MASLD is a chronic liver disease that affects around 30% of the general population in the world, currently with a global prevalence of 25% [5], but it is estimated to grow to 55.7% in 2040, with the greatest increases expected to occur in women and smokers [6]. Overall, the alarming rate of its increase represents a challenge to the prevention and treatment of this disease. Also, the prevalence of MASLD increases with obesity, and is associated with metabolic alterations like type 2 diabetes, dyslipidemia, and metabolic syndrome (MetS) [7]. Another factor that critically influences MASLD prevalence and development is age, since with aging, the liver suffers structural and functional alterations that lead to the impairment of hepatic functions [8]. Although MASLD could appear at any age, in general, the prevalence of MASLD increases with age and older people with MASLD have a greater prevalence of liver fibrosis with a higher risk of mortality compared to younger patients with MASLD [9,10]. However, some studies have also reported that when studying MASLD prevalence and age, the prevalence reaches a peak and then starts to decline as the age increases [11].

Gender has also a great impact on MASLD incidence, clinical manifestations, and development. In general terms, men have a higher MASLD prevalence in fertile age and suffer more severe liver fibrosis. On the other hand, once MASLD appears, women display faster development, with increased inflammation and fibrosis [12]. During reproductive age, women have a significant lower risk of developing MASLD in comparison to men, but once they reach menopause, the prevalence of developing MASLD increases and is comparable to men’s prevalence [13]. Furthermore, postmenopausal women display an increased risk of severe liver inflammation and fibrosis compared to premenopausal women, with a similar severity as men [14]. Although there are several causes that can influence these sex differences, sex hormones have been suggested to play a key role in the development of MASLD, since they regulate lipid and glucose metabolism in the liver. Indeed, the deficiency of estrogen that occurs during menopause increases the risk of MASLD and hepatic fibrosis in women [15,16]. Since aging and menopause are critical factors that influence MASLD progression, the characterization of the molecular mechanisms that favors severe MASLD development during postmenopausal period is critical for finding adequate treatments.

Currently, there are no specific pharmacological treatments approved for MASLD. For this reason, lifestyle modifications such as weight loss, diet modification, and regular physical activity are the first and main approaches in the management of MASLD [17]. Several preclinical and clinical studies have shown the beneficial effects of omega-3 polyunsaturated fatty acids (n-3 PUFA), such as eicosapentaenoic acid (EPA) and docosahexaenoic acid (DHA), on improving MASLD. A previous study from our group showed that long-term DHA supplementation has relevant anti-steatotic effects in a MASLD model of aged obese female mice [18]. Similarly, another study showed that DHA supplementation in obese mice prevented lipid accumulation in the liver and increased fatty acid oxidation [19]. In clinical studies, randomized control trials have proven that n-3 PUFA supplementation significantly reduced liver steatosis and improved serum lipid profiles [20], reducing the incidence of liver disease [21]. Furthermore, in a pilot study, DHA supplementation not only improved fatty acid metabolism in the liver but also hepatic insulin sensitivity [22]. Considering all these results, n-3 PUFA diet supplementation has been proposed as a viable option in the prevention/treatment of MASLD [23].

Although the benefits of DHA are well established, the mechanisms underlying these effects remain largely unknown; therefore, the aim of this study was to characterize the transcriptomic changes in liver underlying the beneficial effects of DHA supplementation on MASLD progression in aged obese female mice.

## 2. Results

**Figure 1:**
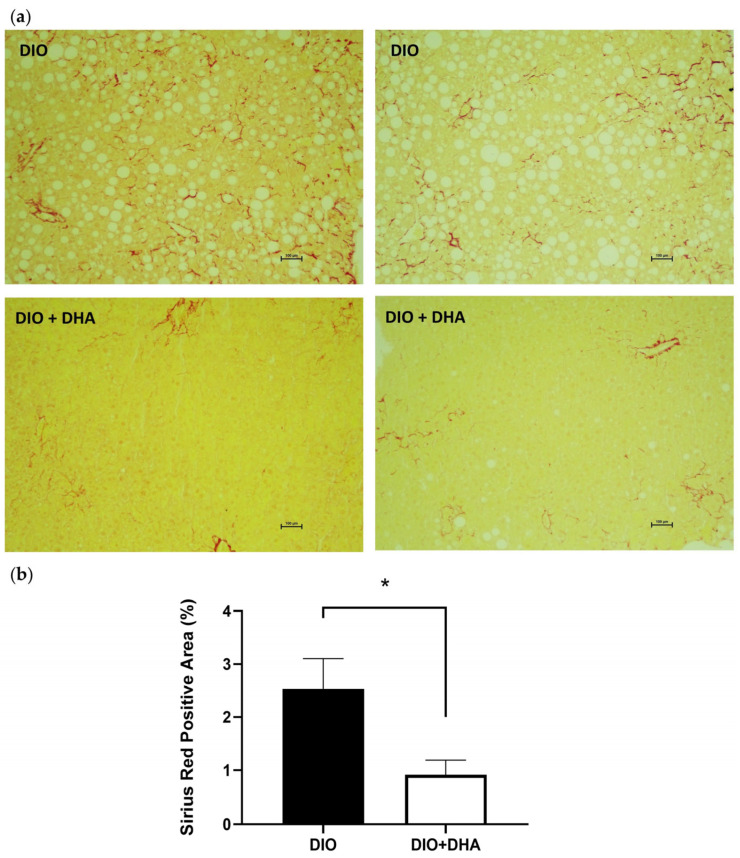
Effects of long-term DHA supplementation on liver fibrosis in aged obese female mice: (**a**) Representative liver histological cross-sections with collagen staining (Sirius Red) from 18 month-old diet-induced obese (DIO) and DIO + DHA mice. (**b**) Quantification of Sirius Red-positive areas (*n* = 5). * *p* < 0.05.

As previously described in other studies from our group, DHA supplementation in diet-induced obese (DIO + DHA) mice did not significantly affect body weight or body composition compared to a diet-induced obese (DIO) group fed only a high fat saturated diet (HFD) [18,24,25]. However, it significantly improved the serum lipid profile by reducing total and LDL-cholesterol levels [26]. Additionally, long-term DHA treatment had beneficial effects on liver health, including lower alanine aminotransferase (ALT) levels, reduced liver weight, and decreased hepatic triglyceride content [18], suggesting a delay in MASLD progression in 18-month-old obese female mice (Table S1). Moreover, red Sirius staining and quantitative analysis of histological images showed that the long-term partial replacement of saturated fat with DHA did not only prevent liver steatosis but also the hepatic fibrosis observed in the aged DIO female mice (Figure 1). Interestingly, all these effects of the DHA treatment on MASLD markers were observed in the absence of significant effects on food intake [25] or body composition (Table S1), suggesting a direct effect of the n-3 PUFAs supplementation on liver function.

In order to explore the mechanisms underlying the beneficial effects of DHA supplementation in MASLD, an RNA-seq study was performed to compare the hepatic transcriptome of the DIO and DIO + DHA groups. A comparison of the DIO and DIO + DHA groups revealed clear differences between samples. When visualized in two dimensions through principal component analysis (PCA), the data showed a distinct separation between the two groups forming two well-defined clusters, highlighting clear differences in global gene expression profiles (Figure S1).

**Figure 2:**
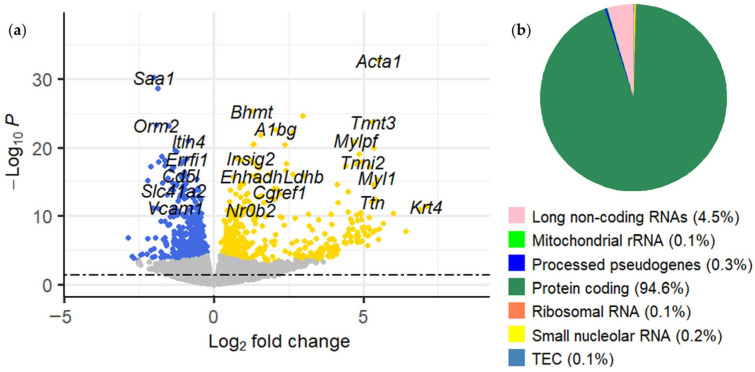
DEGs between the DIO and DIO + DHA groups in liver of obese aged female mice: (**a**) volcano plot showing the distribution of upregulated genes represented as orange dots (Log_2_FC > 0), downregulated genes represented as blue dots (Log_2_FC < 0), and gray dots for genes that are not significant. The dashed line indicates the cutoff for the *p*-value (*P*), set at 0.05; (**b**) classification of DEGs by type.

Differential expression analysis identified 885 differentially expressed genes (DEGs) between the DIO and DIO + DHA groups (using as threshold B > 0). Among these genes, 472 were upregulated and 413 were downregulated (Figure 2a). Expression changes were measured as log_2_ fold change (Log_2_FC), with positive values denoting upregulation and negative values downregulation. Most DEGs were protein-coding (837), while the remainder included 40 long non-coding RNAs, 3 processed pseudogenes, 2 smalls nucleolar RNAs, 1 ribosomal RNA (rRNA), 1 mitochondrial rRNA, and 1 gene of a still experimentally unconfirmed type (TEC) (Figure 2b).

**Figure 3:**
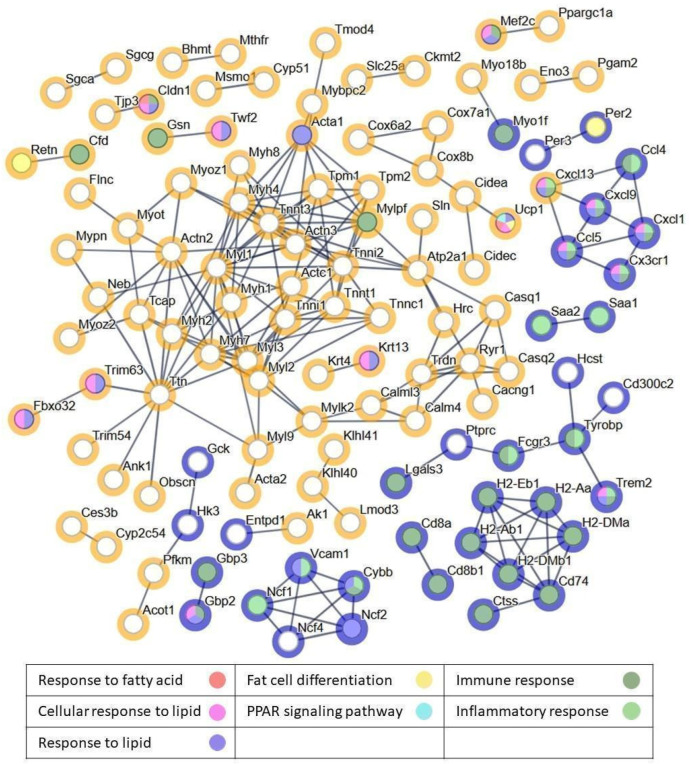
Network of PPI of the top DEGs between the DIO + DHA and DIO groups. Each protein is represented by a circle, the halo of the circle indicates that the RNA is downregulated (blue) or upregulated (orange) and the internal colors show the biological process: response to fatty acid (red), cellular response to lipid (pink), response to lipid (purple), fat cell differentiation (yellow), PPAR signaling pathway (blue), immune response (dark green), and inflammatory response (green).

Analyzing the protein–protein interaction (PPI) networks of the top DEGs that were significant (B > 0) and showed a larger magnitude of change (|Log_2_FC| > 1) revealed a dense network comprising 354 nodes, with almost every gene connected and forming several distinct gene subcommunities (Figure 3). The network demonstrated highly significant connectivity (PPI enrichment *p*-value < 1 × 10^−16^), as 201 interactions were observed compared to the 27 edges expected by chance, supporting functional relationships among the proteins rather than random connectivity. This analysis uncovered interactions among proteins involved in biological processes such as response to fatty acid, cellular response to lipid, response to lipid, fat cell differentiation, PPAR signaling pathway, immune response, and inflammatory response. These findings highlight the importance of lipid and fatty acid metabolism, along with inflammatory responses, in the beneficial effects associated with DHA supplementation.

**Figure 4:**
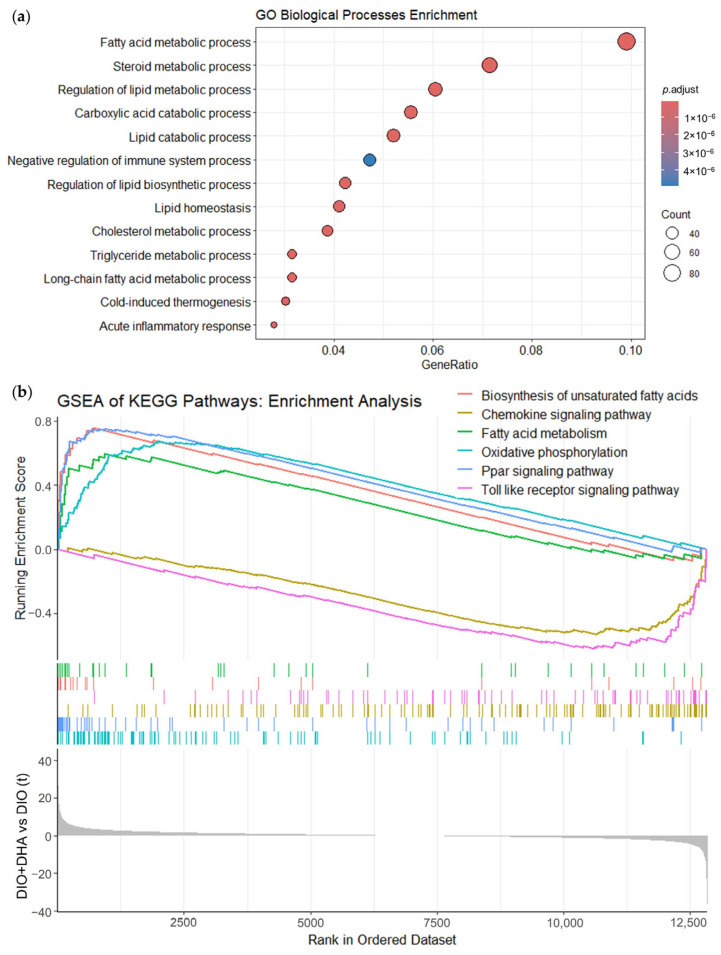
Functional analysis of biological processes and pathways significantly modified by DHA treatment in aged obese female mice: (**a**) GO analysis of biological processes; (**b**) GSEA of KEGG pathways.

To gain a systematic understanding of the biological processes and pathways potentially affected by long-term DHA supplementation, we performed Gene Ontology (GO) enrichment and KEGG pathway analysis. GO analysis of DEGs identified a total of 1182 significantly enriched biological processes (*p*.adjust < 0.05), with fatty acid metabolic processes being the most significant (*p*.adjust = 9.38 × 10^−34^). Notably, other enriched processes included the regulation of lipid metabolic process, steroid, and cholesterol metabolic processes and the negative regulation of immune system process and acute inflammatory response (Figure 4a). These findings indicate that DHA supplementation significantly modulates multiple biological pathways related to fatty acid and lipid metabolism, as well as their regulatory mechanisms, and the immune system. Similarly, Gene Set Enrichment Analysis (GSEA) of KEGG pathways revealed 44 significantly enriched pathways (*p*.adjust < 0.05). Particularly, pathways such as biosynthesis of unsaturated fatty acids, fatty acid metabolism, oxidative phosphorylation, and PPAR signaling were found to be enriched in upregulated genes in response to DHA supplementation. In contrast, pathways enriched in genes downregulated by DHA were primarily related to inflammation and immune response, including those involved in chemokine signaling and the Toll-like receptor signaling pathway (Figure 4b).

**Figure 5:**
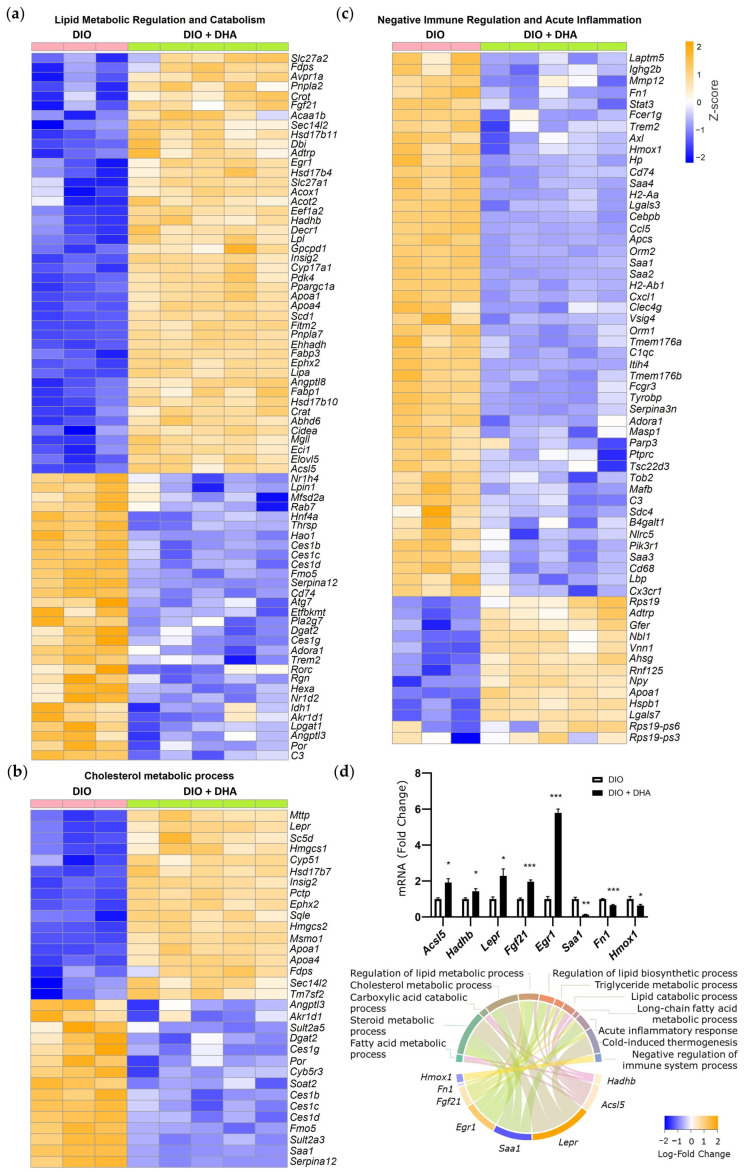
Effects of long-term DHA supplementation on the liver transcriptomic profile of aged obese female mice: (**a**–**c**) heatmap of normalized gene expression profiles (Z-score) for genes related to lipid metabolic regulation and catabolism (**a**), cholesterol metabolic process (**b**), and negative immune regulation and acute inflammation (**c**) between the DIO (pink) and DIO + DHA groups (green) in liver; (**d**) GO terms with Log-FoldChange by RNA-seq and mRNA expression by real-time PCR of *Acsl5*, *Hadhb*, *Lepr*, *Fgf21*, *Egr1*, *Saa1*, *Fn1*, and *Hmox1*. Data values are normalized to the DIO group and expressed as mean ± SEM, *n* = 5–6 animals/group. * *p* < 0.05, ** *p* < 0.01, *** *p* < 0.001 vs. the DIO group.

To examine the expression patterns of genes related to lipid metabolic regulation, cholesterol metabolic process, and immune regulation and inflammation, heatmaps were generated across all samples (Figure 5a–c). Gene expression values were transformed into Z-scores for each gene, representing the number of standard deviations a gene’s expression in a given sample deviates from its mean across all samples. Positive values indicate expression above the gene’s average, while negative values indicate expression below the average, allowing direct comparison of relative expression patterns across genes and samples.

The heatmap of DEGs involved in lipid metabolic regulation and catabolism showed an upregulation of several genes involved in fatty acid oxidation in the DIO + DHA group, such as the *peroxisome proliferator-activated receptor gamma coactivator 1-alpha* (*Ppargc1a*) and its downstream target *acyl-CoA synthetase long chain family member 5* (*Acsl5*), which was also confirmed by qPCR. Similarly, *hydroxyacyl-CoA dehydrogenase trifunctional multienzyme complex subunit beta* (*Hadhb*), a subunit of the *mitochondrial trifunctional protein* (*MTP*), showed higher expression following DHA treatment. DHA supplementation also increased the expression of *fibroblast growth factor 21* (*Fgf21*) and its downstream target *early growth response 1* (*Egr1*), validated by qPCR (Figure 5a,d).

Conversely, genes associated with lipogenesis, including *diacylglycerol acyltransferase 2* (*Dgat2*) and members of the *carboxylesterase 1* (*Ces1*) family including *Ces1b*, *Ces1c*, and *Ces1d*, were downregulated in DHA-supplemented mice (Figure 5a,d). DHA also modulated hepatic cholesterol metabolic processes. Notably, as observed through RNA-seq and validated by qPCR, DHA treatment increased the expression of *leptin receptor* (*Lepr*) (Figure 5b,d).

Finally, analysis of DEGs involved in the inflammatory/immune response revealed the downregulation of genes such as *serum amyloid A* (*Saa*) and *fibronectin 1* (*Fn1*) in DHA-supplemented mice (Figure 5c,d). Additionally, *heme oxygenase 1* (*Hmox1*) expression was decreased in response to DHA (Figure 5c,d).

**Figure 6:**
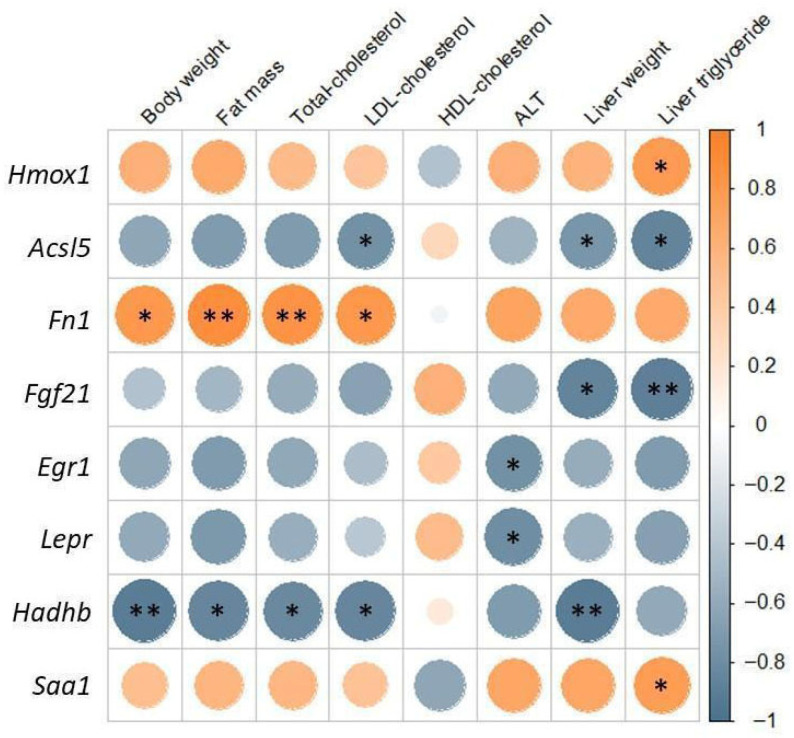
Analysis of the correlation between changes in gene expression and body composition, lipid metabolism, and MASLD biomarkers. Correlation coefficients range from −1 to 1, indicating both the direction and strength of the association: values close to +1 denote a strong positive correlation (higher gene expression associated with higher variable values), whereas values near −1 indicate a strong negative correlation (higher gene expression associated with lower variable values). * *p* < 0.05, ** *p* < 0.01.

All the previously mentioned genes were validated by qPCR. Pearson correlation between RNA-seq and qPCR expression levels for the eight selected genes was high (r = 0.838, *p* = 0.009), demonstrating a strong concordance between both methods (Figure S2). In addition, association analyses were performed between the expression levels of these genes and several parameters related to fatty liver, body composition, and lipid metabolism biomarkers. The analysis revealed that genes involved in fatty acid oxidation, *Acsl5* and *Fgf21*, showed significant negative correlations with liver triglyceride levels and liver weight, the latter also showing a negative association with *Hadhb*. Notably, *Hadhb* also exhibited significant negative correlations with body weight, fat mass, total cholesterol, and LDL-cholesterol, a pattern similarly observed for *Acsl5*. Moreover, *Egr1* and *Lepr* displayed negative correlations with ALT activity. Conversely, significant positive correlations were found between *Fn1* and body fat mass, total cholesterol, and LDL-cholesterol. In addition, the expression of inflammation-related genes *Saa1* and *Hmox1* had a positive correlation with liver triglyceride content (Figure 6).

## 3. Discussion

In agreement with previous studies from our group, the current findings further support that long-term partial replacement of dietary saturated fat with DHA confers direct hepatoprotective effects—improving circulating lipid profile, attenuating steatosis and fibrosis, and lowering markers of liver injury—independently of changes in body weight, body composition, food intake, or insulin sensitivity markers [18,24,25,26]. This supports the potential of n-3 PUFA supplementation as a strategy to slow MASLD progression in obesity and aging.

The main goal of the present study was to characterize the transcriptomic changes that occur in the liver underlying the beneficial effects of long-term DHA supplementation on MASLD progression in a model of aged obese female mice. Our results provided a deep view of the metabolic pathways affected by DHA in MASLD development, and its relevance as a therapeutic approach in combating the disease.

The RNA-seq analysis of liver samples indicated that DHA treatment significantly altered the expression of 885 genes, with a strong biological connection observed among their encoded proteins. Several significantly modified biological processes were related to lipid and fatty acid metabolism, with the fatty acid metabolic process being the most significant and exhibiting the largest number of DEGs, highlighting the importance of this pathway in the beneficial effects of DHA in mitigating MASLD progression. Enriched pathways among upregulated genes included oxidative phosphorylation, PPAR signaling, biosynthesis of unsaturated fatty acids, and fatty acid metabolism, while downregulated genes were predominantly associated with inflammatory and immune responses.

One of the main characteristics of MASLD is liver free fatty acid (FFA) accumulation, mainly in the form of triglycerides, which is the result of an unbalance between fatty acid generation (by de novo synthesis or blood uptake) and its elimination via mitochondrial beta oxidation or export [27,28]. Our novel transcriptomic data further support our previous observations suggesting that the anti-steatotic action of long-term DHA supplementation seems to be mediated by the downregulation of the expression of relevant genes involved in hepatic lipogenesis regulation such as *Dgat2*, *Scd1*, and *Srebp1c*, and the upregulation of those involved in fatty acid oxidation, such as *Acox* [18]. Indeed, the most significantly modified biological process that the GO analysis found is fatty acid metabolism, clearly supporting that DHA is exerting its beneficial effect by readjusting the free fatty unbalance inside hepatocytes. In this regard, we found that DHA supplementation enhances fatty acid beta oxidation inside the hepatocytes by upregulating several genes in this pathway, and thus reducing FFAs accumulation. One of the DEGs, whose expression is augmented, is *Hadhb*, one of the subunits of the MTP, an enzymatic complex that catalyzes the three last steps of long chain fatty acid beta oxidation. This complex is formed by four alpha subunits (encode by *Hadha* gene) and four beta subunits (encode by *Hadhb*), and alterations in *Hadhb* gene have been linked to MTP deficiency [29,30,31]. Precisely, a heterozygous *MTP* defective mice model showed reduced fatty acid oxidation, which promoted hepatic steatosis and the development of MASLD [32], proving the critical role of this complex in lipid metabolism. Furthermore, a disruption in the *Hadhb* gene had been linked with acute fatty liver of pregnancy, which was produced by altered mitochondrial fatty acid oxidation due to fetal MTP deficiency [33]. Finally, the overexpression of the *Hadha* gene in a mice model reduced hepatic steatosis by diminishing oxidative stress and lipid accumulation in the liver [34].

Another gene related to fatty acid metabolism whose expression was significantly increased by DHA is *Acsl5*. This isoform participates in the binding of CoA to long-chain fatty acids, which then can be used for lipogenesis or fatty acid beta oxidation. In mice liver, *Acsl5* seems to mainly direct fatty acids for lipogenesis, but under particular conditions, it also promotes beta oxidation [35]. For example, oncostatin M promotes *Acsl5* transcription in livers of hamsters, leading to a reduction in triglycerides by enhanced fatty acid beta oxidation, and Sirtuin 6 activates ACSL5, increasing hepatic fatty acid oxidation and suppressing MASLD [36,37]. DHA may have a similar effect promoting fatty acid beta oxidation and preventing MASLD progression. DHA also downregulated several genes involved in the cholesterol metabolic process and hepatic de novo lipogenesis. This is the case of *Dgat2*, whose hepatic deficiency in a murine MASLD model has been shown to lower fat accumulation by reducing triglyceride synthesis and de novo lipogenesis [38,39]. DHA supplementation downregulated hepatic *Ces1* expression. The role of CES1 in MASLD is complex and controversial. Some studies have suggested that the overexpression of *Ces1* in the liver has been shown to lower hepatic triglycerides and plasma glucose levels, while the knockdown of *Ces1* increases hepatic triglycerides and plasma cholesterol levels [40]. However, other studies have shown that the attenuation of CES1 activity has a beneficial effect on hepatic lipid metabolism and MASLD progression [41].

Our current results also showed that *Fgf21* was significantly upregulated in the DIO + DHA group, suggesting that the beneficial effects of this n-3 PUFA on age and obesity-related MASLD could be mediated, at least in part, by *Fgf21* upregulation. Interestingly, a study on the effect of fish oil (FO) supplementation in the liver found that the beneficial actions of FO in reducing markers of hepatic lipid accumulation and liver inflammation were blunted in the *Fgf21* knockout mice, suggesting a role for FGF21 in mediating the beneficial effects of FO supplementation on MASLD [42]. Another study also found that the overexpression of *Fgf21* may be able to activate fatty acid oxidation to ameliorate ER stress in the liver and intercept steatohepatitis and inflammatory stress damage [43]. Paradoxically, different studies have described an increased expression of FGF21 in the liver and white adipose tissue of DIO mice and obese humans, as well as in conditions such as hypertension, atherosclerosis, and MASLD [42,44,45]; additionally, ER stress in the liver has also been shown to be a contributor to FGF21 expression, which may activate mechanisms such as augmented ketogenesis, gluconeogenesis, and lipolysis to regulate the liver’s homeostasis [46]. Furthermore, the theory of “FGF21 resistance” has been proposed, a situation in which the inflammation and metabolic stress may reduce the expression of ß-KLOTHO and/or FGFR1—the receptor complex of FGF21—and impair FGF21 signaling pathway, and therefore only an overexpression of *Fgf21* would be able to overcome the situation [42,44,45,47]. In this context, not only should the expression of *Fgf21* be considered in the study of metabolic diseases, but also its sensitivity and receptor complex. Our RNA-seq data showed that DHA supplementation did not modify the expression of *Fgfr1* or *ß-Klotho*. However, the expression of *Egr1*, a target gene of FGF21 [48], was upregulated in DIO + DHA mice in comparison to DIO mice. EGR1 is a rapid-response transcription factor highly expressed in the liver and involved in its metabolism. *Egr1* has also been described in the regulation of adipogenesis, cell growth, survival, proliferation, and apoptosis—critical in the liver’s recovery and regeneration and an important modulator of insulin and cholesterol metabolism in the liver [49]. Notably, EGR1 has also been related to MASLD [50]. A study on the exogenous administration of FGF21 found that *Egr1* gene expression was greatly increased in lean mice, but in obese mice, this was not evidenced possibly due to the “FGF21 resistance” theory, as a result of the deleterious effect on the signaling of FGF21 [51].

In our study, the upregulation of *Egr1* mRNA expression in the DIO + DHA group occurs in parallel to the upregulated expression of *Fgf21*, which may indicate that the expression of both genes is correlated, and also with the DHA supplementation. Moreover, insulin resistance (IR) is an important risk factor in the development of MetS, as well as MASLD [18]. A study showed that the transcriptional regulation of EGR1 could help to regulate steatosis in the liver; however, in patients with IR, the expression of EGR1 was shown to be downregulated, therefore promoting the development of steatosis and MASLD [50]. In accordance with the studies described previously, the upregulation of *Egr1* in our study may induce protective effects against steatosis and inflammation in the liver and improve its activity in carbohydrate and fatty acid metabolism. Our current data suggests that local upregulation of FGF21 signaling in the liver may be involved in the beneficial effects of DHA on MASLD. However, it is important to mention that the regulation of *Fgf21* mRNA expression by DHA supplementation seems to be tissue-specific. Indeed, previous studies of our group have shown that, contrary to what is observed in the liver, long-term feeding with the DHA-enriched high fat diet downregulates *Fgf21* mRNA levels in brown adipose tissue and in subcutaneous white adipose tissue [25,26]. To obtain a better insight into the potential involvement of FGF21 in the systemic effects of DHA supplementation, we measured the circulating levels of FGF21 and found no significant changes between the DIO and DIO + DHA groups (Figure S3). These data suggest that although the liver has been described as the main source of circulating FGF21, in obese aged mice, the contribution of expanded adipose tissue could also be relevant. In summary, our current transcriptomic data suggest a role of hepatic FGF21 signaling in the beneficial effects of DHA on MASLD progression in obesity and aging. However, further studies at the protein level are encouraged to better characterize the modulation of the FGFR1/β-Klotho complex sensitivity and downstream pathway activation.

Leptin is an adipokine that has different effects on MASLD progression: in the early stages of the disease, it has a protective role against steatosis by preventing hepatic lipid accumulation, but as the disease progresses, leptin could have a proinflammatory and profibrogenic role [52,53]. The initial anti-steatotic effect of leptin is due to an upregulation of genes involved in fatty acid beta oxidation, a downregulation of lipogenesis, and an increase in triglyceride export from the liver as very-low-density lipoproteins [53]. It has been shown that diet-induced obesity reduced the expression of leptin receptor in the liver of rats, whilst the upregulation of hepatic leptin receptor induced by metformin reduced lipogenic gene expression and decreased hepatic triglyceride content, alleviating liver steatosis [54]. In the present study, a significant upregulation of *Lepr* was found due to DHA supplementation, which may increase leptin signaling, preventing lipid accumulation in the liver.

Inflammation is a key condition in the development/progression of MASLD, which is characterized by an increased production of proinflammatory cytokines within the liver or by other tissues and reduced anti-inflammatory factors like adiponectin [55]. Previous studies of our group have shown that long-term DHA supplementation reduced the expression of key inflammatory markers in the liver such as the *cytokine tumor necrosis factor alpha* and the *toll-like receptor 4* [18]. Our novel transcriptomic data further support the ability of dietary DHA to attenuate the liver inflammation associated with MASLD concurrent to obesity and aging. Indeed, the GSEA of KEGG pathways revealed that in the DHA-supplemented group, the pathways enriched in downregulated genes were primarily related to chemokine signaling and the Toll-like receptor signaling pathway. Moreover, GO enrichment analysis identified that DHA downregulates hepatic genes involved in the acute inflammatory response. In this regard, we found that DHA supplementation produced an important reduction in *Saa1*, that encodes for an acute phase protein that is mainly produced by the liver in response to tissue damage, inflammation, and infection [56]. *Saa1* has been associated with IR in MASLD, as well as with progression from MASLD to MASH [57]. It has been reported that the levels of this protein are elevated in the liver of mice with MASLD, and that the overexpression of *Saa1* in hepatocytes resulted in lipid accumulation in the liver. Furthermore, in a fatty liver mice model, the knockdown of hepatic *Saa1* reduced inflammation and hepatic steatosis [58]. Similar results were found in another study, where silencing the in vivo expression of *Saa1* reduced platelet aggregation and inflammatory cell recruitment in the liver of mice with MASLD [59]. We have previously reported that the ability of chronic DHA supplementation to reduce inflammatory markers in these aged obese female mice was also observed in other key metabolic tissues such as brown and white adipose tissue and muscle [24,25,26].

Another important feature in MASLD promotion is oxidative stress, which is the result of an imbalance between reactive oxygen species production and antioxidant defenses, and is one of the principal drivers in the progression from liver steatosis to steatohepatitis [60]. Oxidative stress causes DNA, lipid, and protein oxidation, promoting proinflammatory and profibrotic signaling pathways. In fatty liver, the excessive FFA accumulation and beta oxidation promotes mitochondria impairment, which in turn increases reactive oxygen species production [61]. In this context, *Hmox1* is an antioxidant enzyme that metabolizes the heme group and whose upregulation is considered an indicator of oxidative stress. *Hmox1* not only acts as a marker of oxidative stress but also has immunomodulatory functions [62]. It has been shown that in patients with steatohepatitis, *Hmox1* expression is augmented and the increase in its expression reflects the progression of the disease [63]. Therefore, the significant reduction in *Hmox1* mRNA expression, induced by long-term DHA supplementation, suggests a reduction in the generation of reactive oxygen species and may protect the liver against oxidative stress. In consequence, *Hmox1* decrease after DHA supplementation could be associated with the lower activation of the immune system, indicating a reduction in cellular stress and inflammation.

During the development of MASLD, the situation of oxidative stress and chronic inflammation would eventually lead to liver fibrosis. Inflammation will result in hepatic injury and death of hepatocytes and apoptosis, which will activate the hepatic progenitor cells and hepatic stellate cells, resulting in a profibrogenic response [64]. This response consists of the synthesis of extracellular matrix components, like collagen fibers and fibronectin, to repair the tissue injury. RNA-seq analysis showed no significant differences in the mRNA expression levels of collagen; however, DHA supplementation induced a significant reduction in *Fn1* in the liver, which has been shown to play a key role in liver fibrogenesis [65], supporting its involvement in the DHA-induced reduction in liver fibrosis observed by Sirius Red staining.

It is important to mention that although DHA is likely to be the main contributor to the beneficial actions observed on MASLD progression, the DHA-rich n-3 PUFA concentrate used to replace part of the saturated fat of the HFD also contained small amounts of EPA and other n-3 PUFAs (see Material and Methods Section 4). Therefore, we cannot rule out some small contribution of EPA or the other n-3 PUFAs to the described beneficial effects on MASLD.

### Limitations of the Study and Future Directions

The current study provides novel complementary information regarding the transcriptional mechanisms involved in the beneficial effects of long-term DHA supplementation on MASLD progression in aged obese female mice. However, some limitations should be noted. The inclusion of a control group fed with a regular chow diet would have been of interest to characterize the transcriptomic alterations induced by the HFD on the liver of the aged obese mice, and the potential reversion by DHA even in the context of a high fat feeding. It would be of interest to measure in future studies the activity of hepatic enzymes involved in lipogenesis and fatty acid oxidation or the fatty acid oxidation flux. Furthermore, it would be of relevance to characterize the cytokine profiling and the histological quantification of immune cell infiltration in the liver. Future studies should also address the effects of DHA supplementation on physical activity and whole-body energy expenditure, and their potential relationship with the metabolic improvements observed at organ/tissue level. To better characterize the potential involvement of FGF21 signaling in the hepatic effects of DHA supplementation, it would be of relevance to carry out studies in liver-specific *Fgf21* knockout mice.

In conclusion, our current study suggests that long-term DHA supplementation ameliorates MASLD progression in aged obese female mice by promoting transcriptomic changes in genes related to lipid metabolism, acute inflammatory response, fibrosis, and the PPAR signaling pathway.

## 4. Materials and Methods

### 4.1. Animal Models and Experimental Design

All animal experiments of this study were approved by the Ethics Committee for Animal Experimentation of the University of Navarra (Protocol 113–15), in accordance with the EU Directive 2010/63/EU, and carried out according to national animal care guidelines (https://www.unav.edu/investigacion/nuestra-investigacion/etica-para-la-investigacion#ceea (accessed on 19 November 2025)). Seven-week-old female C57BL/6J mice were purchased from Harlan Laboratories (Barcelona, Spain) and housed at the animal facilities of the University of Navarra, under a 12 h light–dark cycle at suitable conditions (22 ± 2 °C, and relative humidity, 55 ± 10%), with free access to food and water. After 10 days of acclimation to the new environment, the animals were fed ad libitum for 4 months with HFD containing as energy: 20% proteins, 35% carbohydrates, and 45% lipids (D12451 diet, Research Diets, Inc., New Brunswick, NJ, USA). Then, DIO mice were randomly assigned into two groups: (1) the DIO group continued with the HFD up to 18 months and (2) the DIO + DHA group, which was fed up to 18 months with the HFD containing a DHA-rich n-3 PUFA concentrate obtained from fish oil, replacing 15% wt/wt of dietary lipids (Research Diets Inc., New Brunswick, NJ, USA). The DHA-rich n-3 PUFA concentrate was obtained from Solutex, Zaragoza, Spain (SOLUTEX0063TG, containing 683.4 mg DHA/g, 46.7 mg EPA/g, with a total content of n-3 PUFA* of 838.9 mg/g as triglycerides; * sum of 18:3 n-3, 18:4 n-3, 20:4 n-3, 20:5 n-3, 21:5 n-3, 22:5 n-3, 22:6 n-3). Other analytical data about the quality of oil included the peroxide and anisidine index and the acid value, which were below the recommended maximum values. Since the DHA-rich n-3 PUFA concentrate contained mixed tocopherols (2 mg/g of Covi-ox^®^ T-79EU), the HFD of the DIO group (from month 6 to 18) was also supplemented with the same amount of tocopherols mix [25,66]. The two diets (prepared by Research Diets, Inc., New Brunswick, NJ, USA) were vacuum-sealed in 2.5 kg plastic bags and kept frozen at −20 °C until used to avoid rancidity. The composition of the diet is described in previous articles of our group [18,25,26]. Mice were socially housed (5 mice per cage) and each mouse was identified by marking the tail with a pen marker. Different colors were used for each experimental group. Animals were subjected to these experimental conditions until the age of 18 months was reached. Weight loss exceeding 20%, immobility, cachexia, or an unresponsive response to stimulation were considered humane endpoints for the study. The transcriptomic and validation studies described in this article were performed in a subsample of mice of the OBELEX project (DIO group *n* = 3–6; DIO + DHA group, *n* = 5). This subsample was representative of the whole population. The experiments and outcomes assessment were not blinded. At the end of the experiment, mice were sacrificed after overnight fasting and liver and blood samples were collected. Livers were weighed and a piece of each liver was obtained for histology studies. The rest of liver samples were quickly frozen in liquid nitrogen and stored at −80 °C with serum samples until further analysis.

### 4.2. Body Composition

Before mice sacrifice, whole-animal body composition was measured in live conscious animals by magnetic resonance technology (EchoMRI-100- 700; Echo Medical Systems, Houston, TX, USA), as previously described [26].

### 4.3. Determination of Liver Triglyceride Content

In order to determine lipid content, liver samples (from 100 to 200 mg) were homogenized in phosphate buffer 0.1 M pH = 7–7.4. Lipid extraction was performed using the Folch method and triglyceride content was quantified using Infinity Triglycerides Liquid Stable Reagent (Thermo Electron Corporation, Colorado Springs, CO, USA) according to the manufacturer’s instructions [67]. Triglyceride content was normalized to mg of protein. Protein concentrations were determined by the BCA method following the manufacturer’s instructions (Pierce-Thermo Scientific, Rockford, IL, USA).

### 4.4. Biochemical Analysis

Serum levels of total cholesterol, HDL-cholesterol, and ALT were measured after a 12 h fasting period using a Pentra C200 autoanalyzer according to the manufacturer’s instructions (Roche Diagnostic, Basel, Switzerland). LDL-cholesterol levels were obtained with the Friedewald equation (LDL-cholesterol = Total cholesterol − HDL-cholesterol − triglyceride content/5). FGF21 serum levels were measured by the Milliplex ELISA kit (Merck, Madrid, Spain).

### 4.5. Sirius Red Staining

Liver pieces were fixed in 3.7–4.0% neutral formalin (pH 7.4) for 24 h, dehydrated with 70% ethanol, and embedded in paraffin. Then, 5 µm thick sections were deparaffinized and stained with Sirius Red for 1 h at room temperature. Fibrosis was quantified as the collagen proportional area (CPA), defined as the percentage of tissue stained with Sirius Red, using FIJI (ImageJ, version 1.54p) with a color deconvolution algorithm [68]. For each animal (*n* = 5 per group), three non-overlapping representative fields at 10× magnification were analyzed, excluding vascularized areas. The mean CPA per animal was used as the unit of analysis.

### 4.6. RNA Isolation and Real-Time PCR

Total RNA from liver was extracted with QIAzol Lysis Reagent (Qiagen; Venlo, Limburg, The Netherlands). An RNA purification step was performed to prevent phenol carryover using the RNeasy Mini Kit (Qiagen; Venlo, Limburg, The Netherlands). RNA concentration was determined with the Nanodrop Spectrophotometer ND1000 (Nanodrop Technologies, Inc., Wilmington, NC, USA) and 5 µg of RNA was then incubated with DNase I (Life Technologies, Carlsbad, CA, USA) for 30 min at 37 °C and reverse transcribed to cDNA with the High-Capacity cDNA Reverse Transcription Kit (Applied Biosystems; Thermo Fisher Scientific, Waltham, MA, USA), following the manufacturer’s instructions. Real-time PCR was performed using the Touch Real-Time PCR System (C1000 + CFX384, BIO-RAD, Hercules, CA, USA).

The expression of selected genes was determined using Power SYBR Green PCR Master Mix (BIO-RAD). Primers, obtained from published studies and validated using Primer-Blast web tool (National Center for Biotechnology Information, Bethesda, MD, USA; https://www.ncbi.nlm.nih.gov/tools/primer-blast (accessed on 19 November 2025)), were used at a final concentration of 10 µM. PCR amplification conditions were as follows: initial denaturation at 95 °C for 10 min to activate the polymerase and denature the DNA, followed by 39 cycles of 95 °C for 15 s (denaturation) and 60 °C for 1 min (annealing and extension combined). Details of primer sequences, including the corresponding NCBI gene record and location, along with other relevant information, are listed in Table 1 following MIQE 2.0 guidelines to ensure transparency and reproducibility [69]. *36b4* was used as the housekeeping gene for normalization. The relative expression of the specific genes was determined using the 2^−ΔΔCt^ method [70].

### 4.7. RNA Sequencing

RNA sequencing was performed by adapting the technology of SCRB-Seq to allow for the high cost-efficient multiplexed transcriptome characterization [78]. Briefly, poly-(A)+ RNA was purified using the Dynabeads mRNA DIRECT Purification Kit (ThermoFisher Scientific). poly-(A)+ RNA was annealed to a custom primer (IDT, Coralville, IA, USA) containing a poly-(T) tract, a Unique Molecule Identifier, and a sample barcode. Retrotranscription using Template-switching oligonucleotides was then used to synthetize and amplify 3′UTR enriched cDNA, resulting in barcoded cDNA fragments. Library preparation was performed using the Nextera XT library preparation protocol which introduces i5-P5 and i7-P7 structure for massive parallel sequencing. Quality control was performed following pre-amplification RT and library preparation to ensure quality and length accuracy, as well as to equilibrate sample pooling. Libraries were then sequenced using a NextSeq2000 sequencer (Illumina, San Diego, CA, USA). Then, 5–10 million pair-end reads (Rd1:26; Rd2:80) were sequenced for each sample and demultiplexed using Cutadapt v4. RNA-seq was carried out at the Genomics Unit of the Center for Applied Medical Research (CIMA, Universidad de Navarra).

### 4.8. G/D RNA-Seq Bioinformatic Analysis

RNA sequencing data analysis was performed using the following workflow: (1) the quality of the samples was verified using FastQC v0.11.8 software and the trimming of the reads with trimmomatic [79,80]; (2) alignment against the mouse reference genome (GRCm39) was performed using STAR v2.7.9a [81]; (3) gene expression quantification using read counts of exonic gene regions was carried out with featureCounts [82]; (4) the gene annotation reference was Gencode vM33 [83]; and (5) differential expression statistical analysis was performed using R/Bioconductor [84]. Data are publicly available in the GEO database with the accession number GSE298504.

First, gene expression data was normalized with edgeR and voom [85,86]. After quality assessment and outlier detection using R/Bioconductor [84], a filtering process was performed. Genes with read counts lower than 5 in more than the 50% of the samples of all the studied conditions were considered as not expressed in the experiment under study. LIMMA was used to identify the genes with significant differential expression between experimental conditions [86]. Genes were selected as differentially expressed using a B cutoff B > 0. Further functional and clustering analyses and graphical representations were performed using R/Bioconductor and clusterProfiler [84,87]. The functional analyses included hypergeometric-based GO enrichment analysis and Gene Set Enrichment Analysis (GSEA) with MSigDB C2 collection of gene sets [87,88].

To explore potential PPI, the STRING database (version 12.0) was accessed using the STRINGdb R package, version 2.14.3 (score threshold = 400; species = Mus musculus, NCBI taxonomy ID: 10090) [89]. Protein-coding genes that were significantly differentially expressed (B > 0) and, among these, only those showing a larger magnitude of change (|Log_2_FC| > 1), were mapped to their corresponding STRING identifiers, with unmapped entries excluded. The resulting identifiers were then used to construct a PPI network in the STRING online platform (version 12.0) using default settings: full STRING network, edges displayed as confidence scores, all interaction sources enabled, and a minimum required interaction score of 0.9.

### 4.9. Statistical Analysis

Statistical analyses were performed using GraphPad Prism 9 software (Graph-Pad Software, La Jolla, CA, USA). Data are presented as mean ± SEM. Differences between groups were considered significant at *p*-value < 0.05. Comparisons between groups were analyzed with unpaired Student’s t or Mann–Whitney’s U test after testing for normality with KS normality test. Correlation analysis was conducted using either Pearson correlation coefficient test or Spearman’s rank coefficient test, depending on normality.

## Figures and Tables

**Table 1:** Mouse (*Mus musculus*) primer sequences for SYBR GREEN real-time PCR. Forward (Fwd) and reverse (Rev) primer sequences are shown in 5′ → 3′ orientation, with their nucleotide (nt) positions and amplicon sizes relative to corresponding NCBI mRNA record. Mean C_q_ values ± standard deviation (SD) and literature references are indicated.

Gene (NCBI Record)	Forward (Fwd) and Reverse (Rev) Primer Sequence and Location (5′ → 3′)		Amplicon Size (bp)	Mean C_q_ ± SD	Reference
*Acsl5* *(NM_027976.2)*	Fwd: TCGATGCAATGCCTGCACT Rev: TGCAGGGACTGAAGGCCA	(nt 3008–3026) (nt 3057–3074)	67	22.84 ± 0.56	[71]
*Egr1* *(NM_007913.5)*	Fwd: GTCCTTTTCTGACATCGCTCTGA Rev: CGAGTCGTTTGGCTGGGATA	(nt 582–604) (nt 634–653)	72	23.39 ± 1.30	[72]
*Fgf21* *(NM_020013.4)*	Fwd: CCTCTAGGTTTCTTTGCCAACAG Rev: AAGCTGCAGGCCTCAGGAT	(nt 480–502) (nt 537–555)	76	24.16 ± 0.49	[73]
*Fn1* *(NM_001276413.1)*	Fwd: ATCGCATTGGGGATCAGTGG Rev: CACTGGTCAATGGGGTCACAC	(nt 1685–1704) (nt 1914–1934)	250	18.88 ± 0.38	[74]
*Hadhb* *(NM_001289798.1)*	Fwd: CCCTGGGAGCTGGCTTCTCTGA Rev: CTCAACACCACCAGCCACGACG	(nt 504–525) (nt 622–643)	140	20.37 ± 0.33	[75]
*Hmox1* *(NM_010442.2)*	Fwd: CCCAAAACTGGCCTGTAAAA Rev: CGTGGTCAGTCAACATGGAT	(nt 1247–1266) (nt 1303–1322)	76	23.69 ± 0.49	[76]
*Lepr* *(NM_146146.3)*	Fwd: TCCAGGAGAGATGCTCACACTTT Rev: TGCGTGGAACAGGTTTGAAA	(nt 3394–3416) (nt 3477–3496)	103	30.45 ± 0.72	[77]
*Saa1* *(NM_001403086.1)*	Fwd: TTGTTCACGAGGCTTTCC Rev: TGAGCAGCATCATAGTTCC	(nt 320–337) (nt 422–440)	121	30.26 ± 1.53	[59]
*36b4* *(NM_007475.5)*	Fwd: CACTGGTCTAGGACCCGAGAAG Rev: GGTGCCTCTGGAGATTTTCG	(nt 502–523) (nt 555–574)	73	19.45 ± 0.22	[18]

## Data Availability

Transcriptomic data are publicly available in GEO database with the accession number GSE298504.

## Supplementary Materials

The following supporting information can be downloaded at: https://www.mdpi.com/article/10.3390/ijms262311689/s1.

## Abbreviations

The following abbreviations are used in this manuscript:

MASLD	Metabolic dysfunction-associated steatotic liver disease
NAFLD	Non-alcoholic fatty liver disease
MASH	Metabolic dysfunction-associated steatohepatitis
MetS	Metabolic syndrome
n-3 PUFA	Omega-3 polyunsaturated fatty acids
EPA	Eicosapentaenoic acid
DHA	Docosahexaenoic acid
DIO + DHA	DHA supplementation in diet-induced obese
DIO	Diet-induced obese
HFD	High fat diet
ALT	Alanine aminotransferase
PCA	Principal component analysis
DEGs	Differentially expressed genes
Log_2_FC	Log_2_ fold change
rRNA	Ribosomal RNA
TEC	Type still to be experimentally confirmed
PPI	Protein–protein interaction
GO	Gene Ontology
GSEA	Gene Set Enrichment Analysis
*Ppargc1a*	*Peroxisome proliferator-activated receptor gamma coactivator 1-alpha*
*Acsl5*	*Acyl-CoA synthetase long chain family member 5*
*Hadhb*	*Hydroxyacyl-CoA dehydrogenase trifunctional multienzyme complex subunit beta*
MTP	Mitochondrial trifunctional protein
*Fgf21*	*Fibroblast growth factor 21*
*Egr1*	*Early growth response 1*
*Dgat2*	*Diacylglycerol acyltransferase 2*
*Ces1*	*Carboxylesterase 1*
*Lepr*	*Leptin receptor*
*Saa*	*Serum amyloid A*
*FN1*	*Fibronectin 1*
*Hmox1*	*Heme oxygenase 1*
FFAs	Free fatty acids
FO	Fish oil
ER	Endoplasmic reticulum
IR	Insulin resistance
Fwd	Forward
Rev	Reverse
nt	Nucleotide
