# Supplementary material for: Transcriptomic Changes Underlying the Anti-Steatotic Effects of DHA Supplementation in Aged Obese Female Mice
Source: Int J Mol Sci. 2025 Dec 2;26(23):11689. doi: 10.3390/ijms262311689 (PMC12691985; doi:10.3390/ijms262311689)
Supplement: Supplementary file 1 [file ijms-26-11689-s001.zip › ijms-3702935 Supplementary material.pdf]

Supplementary materials

Transcriptomic changes underlying the anti-steatotic effects of DHA supplementation in aged obese female mice

Álvaro Pejenaute Martínez de Lizarrondo [1,2#], Paula Martín-Climent [1,2,3#], María Martínez-Rubio [1], Jesús Saborido-Gavilán [1,3], Neira Sáinz [1,3], Elisa Félix-Soriano [1], Miriam Samblas [1], Mónica Alfonso-Núñez [1], Elizabeth Guruceaga [4,5], M Pilar Lostao [1,2,3,4], Pedro González-Muniesa [1,2,3,4], and María J Moreno-Aliaga [1,2,3,4,*]

1 University of Navarra, Department of Nutrition, Food Science and Physiology and Center for Nutrition Research, School of Pharmacy and Nutrition, Instituto de Nutrición y Salud (INS), 31008 Pamplona, Spain; apejenaute@alumni.unav.es; pmartinclim@external.unav.es; mmartinezru@alumni.unav.es; jsaboridoga@unav.es; nsainz@unav.es; elisafelix93@gmail.com; msamblas@unav.es; monalnu@hotmail.com; plostao@unav.es; pgonmun@unav.es

2 CIBER de Fisiopatología de la Obesidad y Nutrición (CIBEROBN), Instituto de Salud Carlos III, 28029 Madrid, Spain.

3 University of Navarra, Instituto de Nutrición y Salud (INS), 31008 Pamplona, Spain;

4 IdISNA—Navarra Institute for Health Research, 31008 Pamplona, Spain.

5 Bioinformatics Platform, University of Navarra, CIMA, 31008 Pamplona, Spain.; eguruce@unav.es

* Correspondence: mjmoreno@unav.es; Tel.: +34-948-425-600 (ext. 806558)

Share equal first authorship

Table S1. Effects of long term DHA supplementation on body weight, body composition, food intake, serum cholesterol profile and markers of MASLD in 18-month-old obese female mice.

	DIO	DIO + DHA
**Body weight (g)**	48.7 ± 3.1	44.9 ± 1.6
**Fat mass (%)**	52.6 ± 2.4	50.6 ± 1.6
**Lean mass (%)**	37.5 ± 1.8	37.6 ± 1.4
**Food intake (kcal/day)**	11.7 ± 0.2	11.2 ± 0.2
**Total-cholesterol (mg/dL)**	130.8 ± 10.1	95.8 ± 9.1 *
**LDL-cholesterol (mg/dL)**	68.3 ± 7.8	32.1 ± 5.8***
**HDL-cholesterol (mg/dL)**	49.9 ± 3.0	53.8 ± 4.9
**ALT (U/L)**	172.1 ± 30.6	83.2 ± 2.3 *
**Liver weight (g)**	1.5 ± 0.1	1.2 ± 0.0 **
**Liver triglyceride (mg/g tissue)**	177.5 ± 9.3	100.9 ± 6.5 ***

Data are expressed as mean ± SEM. n = 6 (DIO) or n= 5 (DIO + DHA) animals/group. * $p < 0.05$, ** $p < 0.01$, *** $p < 0.001$ vs DIO group.

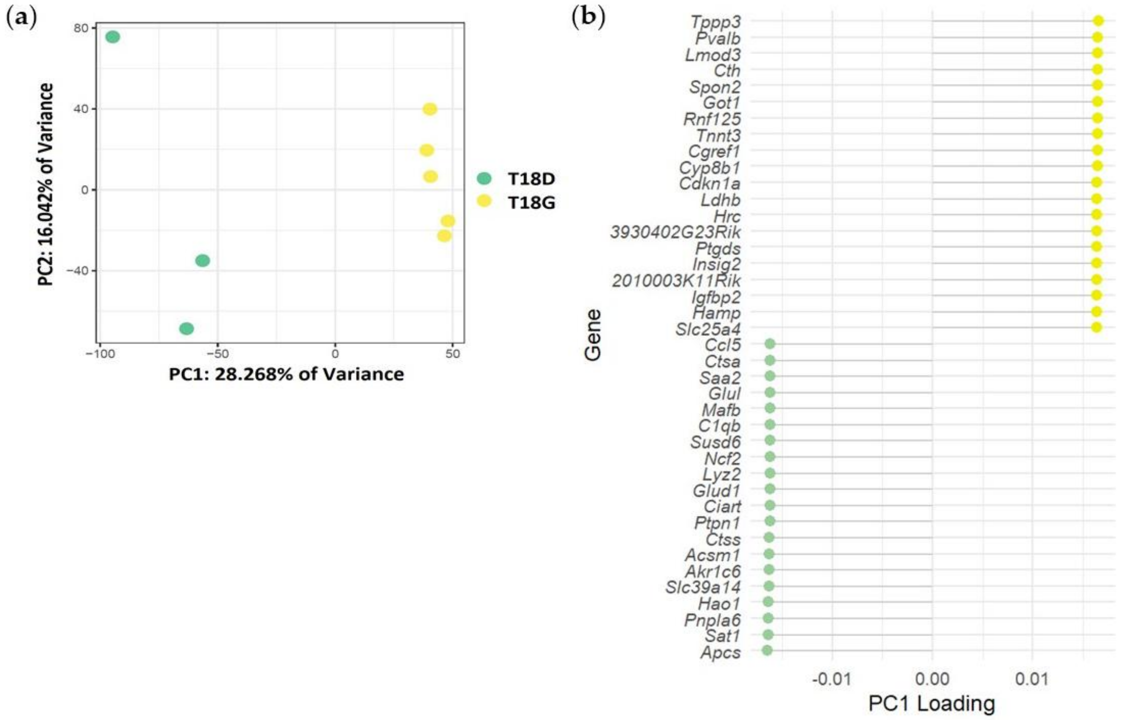

Figure S1. a) PCA analysis of the samples of DIO (green) and DIO+DHA groups (yellow). b) Top Genes Contributing to PC1 that explain 28% of variance.

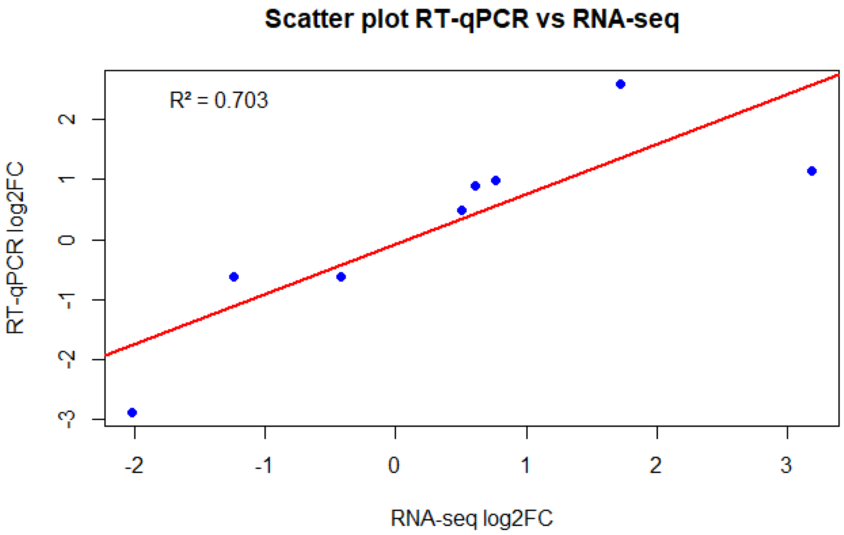

Figure S2. Concordance between RNA-seq and RT-qPCR results. Scatter plot of log2 fold-change (log2FC) values measured by RNA-seq (x-axis) versus RT-qPCR (y-axis) for the 8 selected genes. Each point represents one gene. The red line indicates the linear regression fit. The coefficient of determination (R^2 = 0.70) are shown.

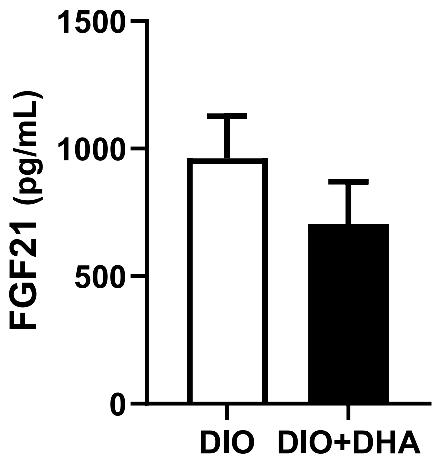

Figure S3. Effects of long-term DHA supplementation on FGF21 serum levels of aged diet-induced obese (DIO) female mice. Data are mean ± SEM (n = 6-5 animals per group)
